# Where Did Vessels Come from? A Study of Pottery Provenance from the Site of Velika Humska Čuka, Serbia

**DOI:** 10.3390/ma18051083

**Published:** 2025-02-28

**Authors:** Maja Gajić-Kvaščev, Ognjen Mladenović, Petar Milojević, Aleksandar Bulatović

**Affiliations:** 1 Vinča Institute of Nuclear Sciences, National Institute of the Republic of Serbia, University of Belgrade, 11001 Belgrade, Serbia; 2 Institute of Archaeology, National Institute of the Republic of Serbia, 11000 Belgrade, Serbia; mladenovic40@gmail.com (O.M.); pertinax1983@gmail.com (P.M.); abulatovic3@gmail.com (A.B.)

**Keywords:** Velika Humska Čuka, elemental composition, EDXRF spectrometry, Early Eneolithic, pottery provenance, Central Balkans

## Abstract

The archaeological materials from the Velika Humska Čuka site on the northern fringe of the Niš Basin in southeastern Serbia were analyzed to reveal the provenance of ceramics and other artifacts. This study focused on the elemental analysis of 61 samples, including local clay pits, potsherds, and whole vessels. Samples were chosen based on stylistic and typological characteristics to distinguish local and “foreign” pottery. Elemental analysis was conducted using energy-dispersive X-ray fluorescence (EDXRF) spectrometry, complemented by principal component analysis (PCA) for data interpretation. Results indicated that the majority of pottery samples, over 80%, were produced using local clay from deposits near the site. However, approximately 20% of the analyzed vessels were made using clay from deposits near the Bubanj site, 8 km south of Velika Humska Čuka. A vessel on a hollow high foot combining stylistic elements of the Bubanj-Hum I group and Early Eneolithic Pannonian groups was made of clay not sourced from any identified local deposits, suggesting its non-local origin. While the predominance of local materials suggests self-sufficient production, the use of non-local clays and stylistic influences highlights long-distance connections and exchanges. The study emphasizes the importance of Velika Humska Čuka in understanding the development of ceramic traditions and the cultural dynamics of the Early Eneolithic in the Central Balkans.

## 1. Introduction

**Figure 1:**
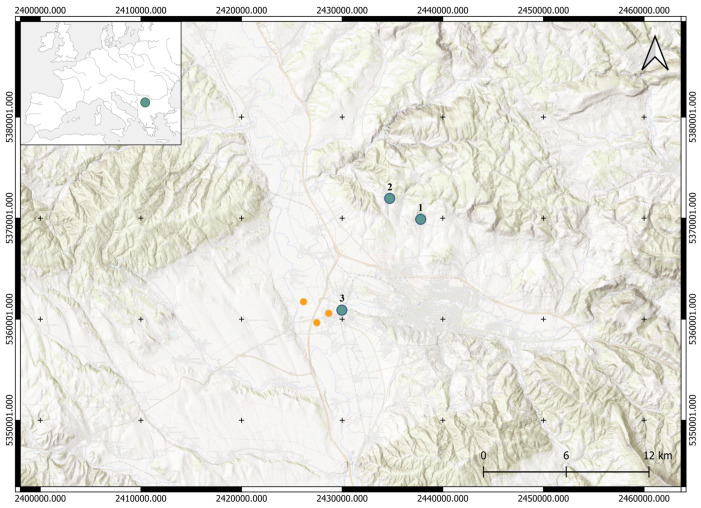
The sites of Velika Humska Čuka (1), Kremenac (2), and Bubanj (3); clay pits near the site of Bubanj—orange dots (Esri Topo Map/Open Topo Map; EPSG: 3857—WGS 84/Pseudo-Mercator; QGIS 3.28. Firenze).

The multilayered site of Velika Humska Čuka is located approximately 10 km east of the South Morava River and 7 km north of the present-day city of Niš, on the northern fringe of the Niš Basin in southeastern Serbia (Figure 1). The site lies on a dominant and hardly accessible elevation that comprises four terraces on different altitudes. In total, the plateau covers an area of 200 × 160 m^2^, with the highest altitude of around 445 m above sea level. The site is surrounded by the Hum River on the northern and western sides and is relatively easily accessible only from the northeastern side. In the north, the site is connected with a smaller elevation, the Mala Humska Čuka [1]. The dominant position that the site holds enables good visual connection with the surroundings, particularly the sites of Bubanj and Kremenac (Figure 1). The site of Kremenac is known as a source of quality raw materials for the production of lithic tools [2].

The first data about the site originates from the beginning of the 20th century and the survey conducted by V. Fewkes. Soon after, the first archaeological excavations were conducted in 1932 and 1933 by V. Grbić and the National Museum in Niš. In 1938, the excavations were continued by B. Gojković. Of particular importance are the excavations in 1956 by M. Garašanin, which served as a basis for the definition of the Bubanj-Hum group that marks the Copper and Bronze Age in this part of the Central Balkans [1,3,4]. Finally, the ongoing excavations were renewed in 2009 by the Institute of Archaeology in Belgrade and the National Museum in Niš [1]. The excavations resulted in the discovery of numerous archaeological features from different prehistoric and historic periods, as the site was settled throughout the Copper Age, Bronze Age, Iron Age, Antique Period, and Medieval Period [1,5]. However, the importance of the site and the neighbouring site of Bubanj is particularly reflected in their potential for the study of the development and chronology of the Early Eneolithic in the region [6].

Due to the multilayered nature of the site, well-defined stratigraphy and archaeological features, and the variety of the material culture, the site of Velika Humska Čuka acted as one of the pillars of the FLOW project (THE FLOW is the acronym for the multidisciplinary project Interactions-Transmission-Transformation: Long-distance connections in the Copper and Bronze Ages of the Central Balkans, which is financed by the ScienceFund of the Republic of Serbia, IDEAS call, Grant no. 7750074; the project runs from 2022 to 2025 and incorporates the joint work of archaeologists, physicists, and chemists). Namely, different categories of the material culture from the site were incorporated into the project, which included the provenance analyses of copper, bronze, obsidian [7], and now ceramic artifacts.

While examining archaeological ceramics, various scientific analytical techniques can be used to reveal their chemical and/or mineralogical composition [8,9,10]. Currently, non-invasive techniques offer more advantages and are more used in practice [11,12,13]. This approach was used in the study to examine the characteristics of materials from which vessels and potsherds were made. The materials were characterized in terms of elemental composition determined using energy-dispersive X-ray fluorescence (EDXRF) spectrometry. By comparing them with the characteristics of clay samples, the usage of local raw materials for production was discussed. It was discovered that a whole assemblage of potsherds of different kinds and periods was locally produced, while certain vessels had a non-similar chemical composition with the local clay. The analytical results confirm the archaeological study of these findings.

## 2. Materials and Methods

### 2.1. Samples

The provenance analyses based on the comparison of elemental compositions were conducted on a total of 61 samples, of which 5 samples originate from local and nearby clay pits, 45 samples originate from targeted potsherds from all prehistoric periods registered at the site, 10 samples originate from Early Eneolithic vessels of unusual stylistic or typological characteristics from different archaeological contexts, and 1 control potsherd. The idea was to compare the elemental composition on a diachronic and cross-cultural scale represented at the site, and hence, the selection of samples from the site was based on those parameters. Further, the choice of samples was based on the stylistic and typological characteristics that indicated both the “foreign” and domestic origin of pottery. The “foreign” pottery was defined by certain forms and ornamentation styles that demonstrate the cultural attribution uncommon for the existing cultural stratigraphy of the South Morava Basin.

**Figure 2:**
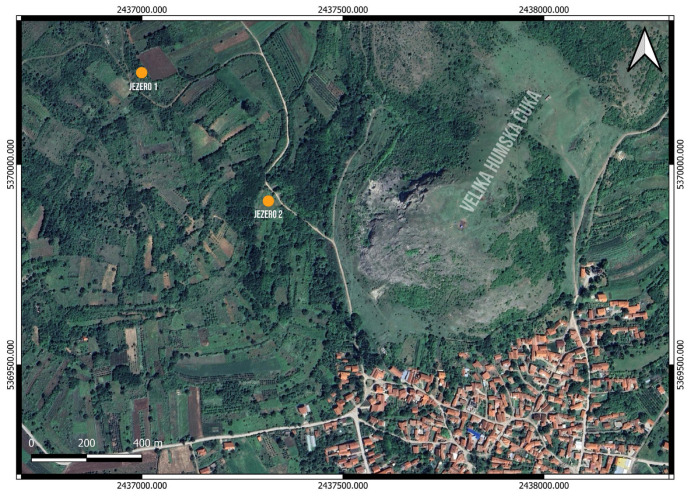
Position of clay pits Jezero 1 (1) and Jezero 2 (2) in relation to the Velika Humska Čuka (Google Satellite; EPSG: 3857—WGS 84/Pseudo-Mercator; QGIS 3.28. Firenze).

Two samples from local clay pits near Velika Humska Čuka, marked as Jezero 1 and Jezero 2, were collected on-field and selected based on communication with the local population of the village of Hum, where the site is located. Location Jezero 1 is located approximately 700 m northwest of the site, and location Jezero 2 is located around 330 m to the west (Figure 2). The sample from location Jezero 1 was used to make two clay tiles for analysis, while Jezero 2 represented a control sample that resembled soil rather than clay. An additional three clay samples from the nearby multilayered prehistoric site of Bubanj (Crepana in Novo Selo, Crepana in Lalinac, and Tri Bresta) were included in the study [14].

**Figure 3:**
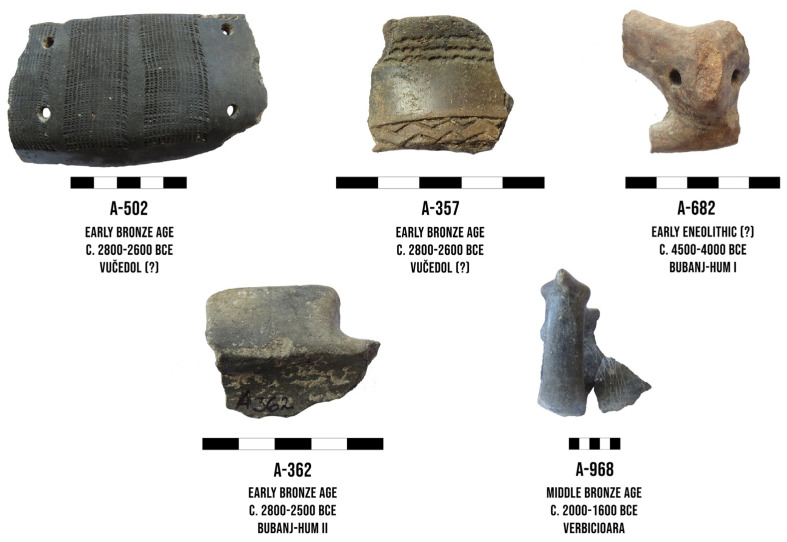
Examples of the potsherds used in the study (each scale is 5 cm).

The 45 samples that are taken from potsherds originate from different archaeological features and the cultural layer at the site (Figure 3). The number of samples covered a wide chronological (c. 4500–1000 BCE) and cultural span, including the Early Eneolithic (Bubanj-Hum I/Bubanj-Sălcuţa-Krivodol complex) (9 samples), Middle Eneolithic (Sălcuţa IV group) (3 samples), Late Eneolithic (Coţofeni-Kostolac group) (4 samples), Early Bronze Age (Bubanj-Hum II, Bubanj- Hum III groups) (10 samples), Middle/Late Bronze Age (Verbicioara group) (8 samples), Late Bronze Age (Brnjica group) (7 samples), and the Transitional Period (Gava-Belegiš II group) (4 samples).

**Figure 4:**
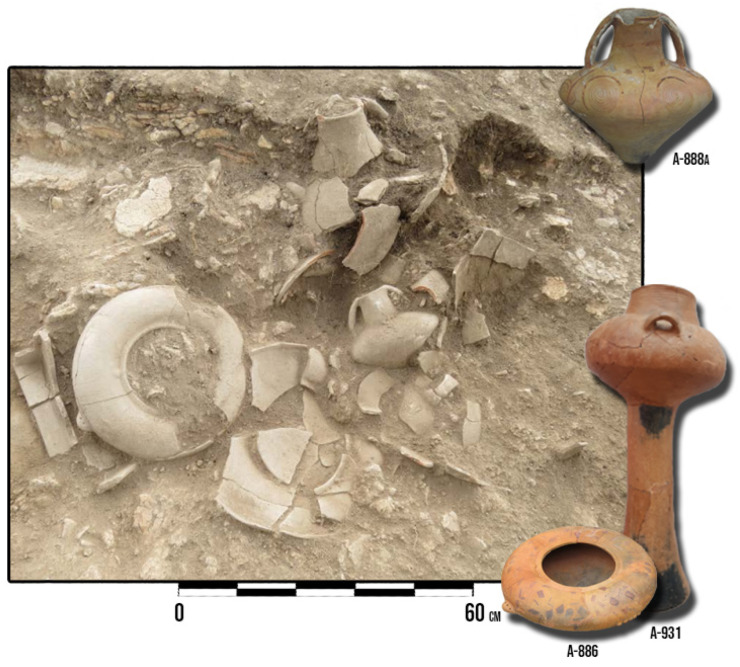
A set of Early Eneolithic vessels in situ during 2018 excavations (7573337.0822, 4804286.2416—coordinate of the central vessel with two handles) (EPSG: 31277—WGS 85/Balkans Zone 7).

The final set of samples is represented by a group of specific lavishly decorated and painted vessels from House 3, a vessel from House 4, and vessels from the pottery accumulation in the trench 1/18 on the southern edge of the site. Most vessels were discovered in connection with Early Eneolithic House 3 in two pits that were dated to the first half of the 44th century BCE. Those vessels were sampled since most of them bear characteristics of the Bubanj-Hum I group, a local manifestation of the Bubanj-Sălcuţa-Krivodol complex of the Central Balkans, yet certain forms indicate the permeation with the Early Eneolithic groups of the Lower Danube Region, such as the Gumelniţa group, or groups from the Pannonian regions, such as the Tiszapolgár and Lasinje groups. Further, the ornamentation of some of the vessels, represented by asymmetrical geometric motifs painted in several different colors (purple, red, white, blue), at the moment, besides a few vessels from nearby Bubanj site, represents a unique manifestation of the Early Eneolithic in the wider region (Figure 4) [1]. Vessels from House 4 and pottery accumulation in trench 1/18 show exclusively stylistic and typological features characteristic of the local Bubanj-Hum I group.

### 2.2. Sample Preparation

The ceramic potsherds were prepared for analysis in a standardized manner for elemental characterization using a handy sander equipped with a diamond blade. Suitable parts of the potsherds were polished to achieve a maximally flat surface and then cleaned. Where possible, the potsherds were polished in one to three different parts. Each potsherd was analyzed at several points, and the average value of collected spectra was used for further analysis [15].

The whole vessels were not treated before the analysis. The suitable fractured parts that remained visible were chosen for the analysis, and care was taken to select the flattest possible.

**Figure 5:**
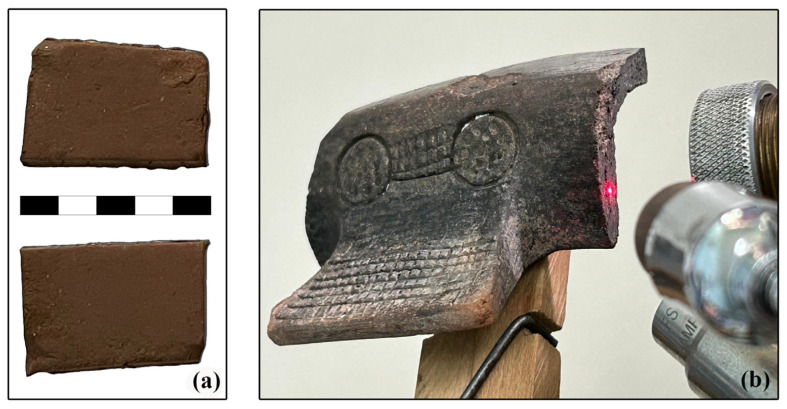
(**a**) Clay tiles from Jezero 1 clay pit and control sample Jezero 2 (scale is 5 cm); (**b**) measurement of an Early Bronze Age potsherd using EDXRF spectrometer.

The clay samples were prepared in the form of tiles with dimensions around 5 × 4 cm and a thickness of 3 cm (Figure 5a). After the clay is dried and impurities are removed, the material is slowly hydrated to form the paste with a suitable consistency for molding. After tile shaping and air drying for several days, they were fired at 400 °C [16]. The tiles were analyzed under the same conditions as the ceramic material to test the assumption about local raw material sources.

### 2.3. Analytical Technique and Data Analysis

The ceramic material was characterized in terms of elemental composition. To establish these results, EDXRF spectrometry was employed for qualitative analysis. An in-house-developed EDXRF spectrometer was used (developed at Vinča Institute of Nuclear Sciences, Figure 5b). The spectrometer has a milli-beam spot of an air-cooled X-ray tube (Oxford Instruments, Scotts Valley, CA, USA, Rh anode, maximum 50 kV, maximum current 1 mA) with a pin-hole collimator. The instrument is equipped with a Si-PIN X-ray detector (6 mm^2^/500 μm, Be window 12.5 μm thickness, with an energy resolution of 160 eV at 5.89 keV). The detector is associated with a DSP (X123, Amptek Inc., Bedford, MA, USA) for spectra acquisition. Two laser pointers, mounted onto the spectrometer head, serve for proper sample positioning. ADMCA software (Amptek Inc., version 1, 0, 0, 16) was used for spectra acquisition and processing. The following parameters, X-ray tube voltage of 40 kV and 800 μA current, without filter, together with measuring time of 120 s, were kept constant during all measurements.

All collected EDXRF spectra were pretreated by the peak balancing procedure (using the original MATLAB (ver. R2023b) code based on peak alignment) to minimize any contributions of the experimental setup. All 61 aligned measurements were organized in a 61 × 2048-dimensional matrix.

Due to the lack of certified reference materials for the quantification of the chemical composition of archaeological ceramic materials, provenance studies are based on the similarities/differences between the analyzed samples and raw materials. To reveal similarities/differences between examined samples, the analytical results were subjected to a multivariate analysis to transform multidimensional space into lower-dimensional space. Principal component analysis (PCA) was chosen for this purpose. It is the most often used unsupervised multivariate technique for dimension reduction [17,18,19]. This technique was chosen for the study because dimension reduction was performed without prior knowledge about the objects’ membership in a specific group. Therefore, the resulting picture of grouping the lower-dimensional space can be put into an archaeological context. The PCA-based dimension reduction was performed on raw spectra containing overall spectral information [20,21].

## 3. Results and Discussion

**Figure 6:**
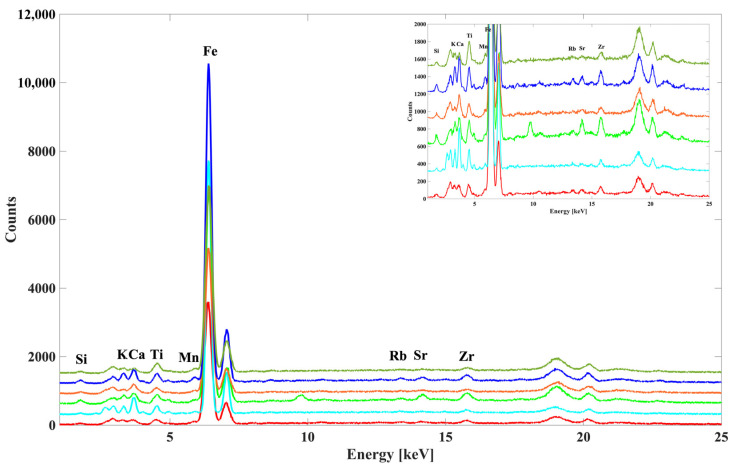
Representative EDXRF spectra collected at vessels, potsherds, and clay samples.

The EDXRF spectra collected on the randomly selected vessels and potsherd body, as well as clay samples, show similar elemental compositions. The Si, K, Ca, Ti, Mn, Fe, Rb, Sr, Y, and Zr are detected in all the measured samples (Figure 6).

**Figure 7:**
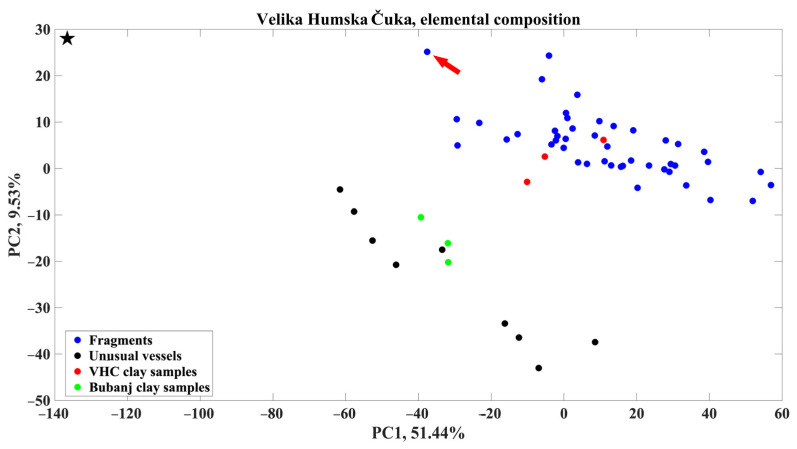
PCA-based dimension reduction.

As we mentioned in the above text, the provenance study was performed based on the qualitative EDXRF results, as its results are comparable to the quantitative ones [14]. The PCA-based dimension reduction, performed on the dataset formed as described above, is shown in Figure 7. During PCA, a new two-dimensional space was created from the initial 2048-dimensional one, following the variance of the dataset. The first two principal components (PC1 and PC2) account for the maximal variance of the initial data. According to the results of the PCA, the first two principal components account for nearly 61% of the maximal variance, giving an acceptable picture of the initial dataset structure in a newly formed space. However, the projection of 61 dataset elements in the PC1–PC2 space shows that two groups are formed and clearly separated, indicating a difference between members in newly formed groups caused by differences in the elemental composition.

The results show that the analyzed potsherds from different prehistoric periods (blue dots) have a similar elemental composition as the clay samples (Jezero 1 and Jezero 2, red dots), indicating the use of local raw materials and local production of ceramic pots for various utilities. As can be seen from Figure 7, the group that contains potsherds is quite coherent. This result indicates continuity in the ceramics production during the long period at the site of Velika Humska Čuka. Only one potsherd, denoted as A-502 (marked with an arrow and shown in Figure 3), deviates slightly from this finding, making the group slightly less coherent. A slight deviation in the elemental composition of the A-502 sample might bear connections with their cultural attribution. Namely, analyzed sherds bear certain ornamental characteristics of the Vučedol group, such as polished surface and ornamentation organized in vertical friezes. The Vučedol group is primarily connected with the territory of Western Balkans, from where it spread towards the Adriatic, Carpathian Basin, and parts of Central Europe [22,23]. However, its penetration towards the east has been recorded on multiple sites, although as an exception rather than a common feature. Interestingly, the Bubanj-Hum II group, which marked the earlier phase of the Early Bronze Age in the region, bears certain ornamental characteristics of the Vučedol group and the Coţofeni-Kostolac group, but analyzed samples of the Bubanj-Hum I group were made by local clay [4,6,24]. It is important to highlight that the absolute and relative chronologies of the given groups overlap in the first quarter of the 3rd millennium BCE [6,25,26,27,28], and hence, such a permeation and continuation of ornamental elements might indicate certain contacts between different yet contemporary communities.

The group formed by the EDXRF measurements on the Early Eneolithic vessels of uncommon characteristics is less coherent than the one comprising the potsherds. The group is divided into two parts. The differences between the members of these two groups might originate even from measuring conditions since the measuring spots were not prepared prior to analysis.

For the complete vessel marked as A-931 (denoted as a star mark in the upper left corner in Figure 7 and shown in Figure 4—bottom right corner), additional consideration is necessary, as it is far from any other sample in the study. The vessel originates from the large pit within House 3, dated to the first half of the 44th century BCE. Sample A-931 is a vessel whose stylistic and typological characteristics of the upper portion, meaning the recipient can be connected with the local Early Eneolithic group (Bubanj-Hum I group). The high hollow foot of the vessel is characteristic of the Pannonian groups of the Early Eneolithic.

As can be seen from Figure 7 (black dots), the vessels seem to be made of raw materials that differ in the elemental composition of local ones. In addition to vessel A-931, the pit contained vessels made from both local (Jezero 1 and 2) and ‘foreign’ clay. This suggests that vessels made from different clays, or those of different origins, were treated similarly within the Early Eneolithic household at the site. This was the reason for introducing the EDXRF measurements of the samples prepared from the clay sampled from the nearby simultaneous archaeological site Bubanj. The introduction of the results of the analyses of clays from deposits in the vicinity of the nearby site of Bubanj (Figure 7, green dots) indicate that their composition almost perfectly matches the composition of unusual vessels from the site of Velika Humska Čuka which differentiate in the PC1-PC2 picture (Figure 7, black dots).

The remaining samples from both the earlier and the later phase of the Early Eneolithic, were most likely made of local clay from the aforementioned clay pits (Jezero 1 and 2, red dots), and though in small number, from the clay from clay pits in the vicinity of Bubanj (Crepana in Novo Selo, Crepana in Lalinac and Tri Bresta, green dots). Hence, there is no emerging pattern regarding the utilization of a specific clay for a certain type of vessel or a certain phase of the Early Eneolithic. Although the number of samples is currently too low for some final conclusions, it can be noticed that the graphite-painted pottery, which is one of the main characteristics of the local Bubanj-Hum I cultural group of Early Eneolithic from the site of Velika Humska Čuka, is made exclusively from local clay. On the other hand, pottery painted in red, purple, white, and other colors, decorated with motifs uncommon for the Bubanj-Hum I group, is made exclusively from clay in the vicinity of the site of Bubanj. These data are not surprising, considering that the pottery of similar stylistic characteristics, painted in different colors and decorated with similar geometric motifs, was recorded sporadically at the site of Bubanj during the excavations in the 1930s [29].

**Figure 8:**
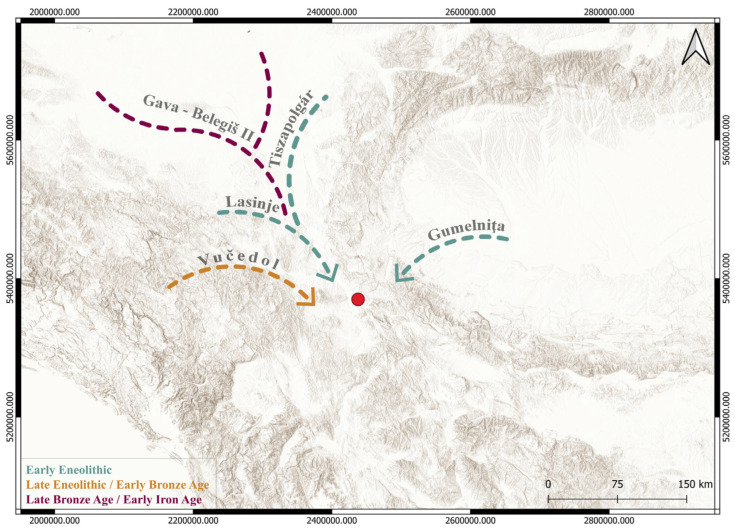
Representation of cultural influences mentioned in the study (Esri Topo Map; EPSG: 3857—WGS 84/Pseudo-Mercator; QGIS 3.28. Firenze).

Interestingly, the samples of vessels which were based on the stylistic and typological characteristics marked as “foreign”, or of northern origin, such as the vessel with the *Scheibenhankel* type of pottery attributed to the late phase of the Early Eneolithic of Oltenia and Banat, vessels with characteristics of the Late Eneolithic Vučedol group, or the Gava-Belegiš II group from the end of the Late Bronze Age [30,31,32,33], were in fact made of local clay, from the clay pits of Jezero 1 and 2. This also illustrates the contacts between the communities of the Nišava Valley and the communities of the Morava Valley and the Pannonian Plain (Figure 8), which resulted in the production of imitations of foreign pottery made of local clay.

## 4. Conclusions

The presented data call for certain conclusions, which are not final due to the upcoming excavations at this site yet provide certain insights into the possible origin of clay for pottery production at the site of Velika Humska Čuka.

Based on the picture of the dataset structure in the PC1-PC2 space following maximal variance among the data (with nearly 61% preserved variance), the following conclusions can be withdrawn. The majority of pottery samples, 45 out of 55 (more than 80%), from different periods of prehistory, and “foreign” or unusual stylistic and typological characteristics were made of local clay from the vicinity of the site (Jezero 1 and 2).

Only 10 samples (less than 20%) were made of clay whose elemental composition matches a different clay deposit, in this case, the deposits in the vicinity of the site of Bubanj, located around 8 km south of Velika Humska Čuka. These vessels originate from three different archaeological contexts, dated to the 44th century BCE. The stylistic common feature of most of these vessels (five examples and all vessels from pits within House 3) is their decoration comprising uncommon geometric motifs and paintings in different colors, similar to the pottery recorded at the site of Bubanj in the 1930s.

This leads to the assumption that those vessels were exchanged as final products with the inhabitants from the site of Bubanj or that the clay was due to the quality brought from those distant deposits.

Such origin of pottery from the site of Velika Humska Čuka indicates intensive contact with the population at the site of Bubanj, whether it was an exchange of goods, seasonal movements between the two sites, or a shared use of clay pits in the vicinity of Bubanj.

On the other hand, the vessel on a hollow high foot (Figure 7, black star—A931), whose recipient is common for the Early Eneolithic Bubanj-Hum I group, and the foot characteristic for the contemporary groups in Pannonia, was made of clay whose elemental composition does not match either of the presented clay deposits and most likely represents a vessel of a non-local origin.

## Data Availability

The original contributions presented in this study are included in the article. Further inquiries can be directed to the corresponding author.
